# Improving preclinical to clinical translation of cognitive function for aging-related disorders: the utility of comprehensive touchscreen testing batteries in common marmosets

**DOI:** 10.3758/s13415-023-01144-x

**Published:** 2024-01-10

**Authors:** Takeshi Murai, Lauren Bailey, Laura Schultz, Lauren Mongeau, Andrew DeSana, Afonso C. Silva, Angela C. Roberts, Stacey J. Sukoff Rizzo

**Affiliations:** 1 Aging Institute, University of Pittsburgh School of Medicine, Pittsburgh, PA USA; 2 Department of Neurobiology, University of Pittsburgh School of Medicine, 514A Bridgeside Point 1, 100 Technology Drive, Pittsburgh, PA 15219 USA; 3 Department of Physiology, Development and Neuroscience, University of Cambridge, Cambridge, UK

**Keywords:** Common marmosets, Cognition, Aging, Alzheimer’s disease, Spatial working memory, Cognitive flexibility

## Abstract

Concerns about poor animal to human translation have come increasingly to the fore, in particular with regards to cognitive improvements in rodent models, which have failed to translate to meaningful clinical benefit in humans. This problem has been widely acknowledged, most recently in the field of Alzheimer’s disease, although this issue pervades the spectrum of central nervous system (CNS) disorders, including neurodevelopmental, neuropsychiatric, and neurodegenerative diseases. Consequently, recent efforts have focused on improving preclinical to clinical translation by incorporating more clinically analogous outcome measures of cognition, such as touchscreen-based assays, which can be employed across species, and have great potential to minimize the translational gap. For aging-related research, it also is important to incorporate model systems that facilitate the study of the long prodromal phase in which cognitive decline begins to emerge and which is a major limitation of short-lived species, such as laboratory rodents. We posit that to improve translation of cognitive function and dysfunction, nonhuman primate models, which have conserved anatomical and functional organization of the primate brain, are necessary to move the field of translational research forward and to bridge the translational gaps. The present studies describe the establishment of a comprehensive battery of touchscreen-based tasks that capture a spectrum of domains sensitive to detecting aging-related cognitive decline, which will provide the greatest benefit through longitudinal evaluation throughout the prolonged lifespan of the marmoset.

## Introduction

For decades, rodents, and in particular mice, have been the primary animal models for studying the biology of aging and neurodegenerative disorders and relatedly cognitive function and impairment. Rodents have provided important insights into the genetics and underlying disease pathophysiology of aging and aging-related disorders and continue to be excellent tools for preclinical in vivo studies, especially those that inform drug pharmacokinetics, safety, and toxicity (Nakai et al., 2021; Poon et al., 2020; Sasaguri et al., 2017; Vitek et al., 2021; Webster et al., 2014). Critically, however, rodents as translational model systems for functional outcome measures, including translating cognitive improvement of potential novel therapeutics have had limited to no success (Drummond & Wisniewski, 2017; Reiss et al., 2020; Sasaguri et al., 2017; Veening-Griffioen et al., 2019; Silverman et al., 2020). While the assays used to measure cognitive endpoints in rodents have their own limitations and are a likely contributing factor to the lack of translational success (Silverman et al., 2020), rodents lack key anatomical brain regions and neurocircuitry involved in higher order cognitive processes. This severely limits their translational potential for modeling specific biological consequences as well as their utility for predicting specific cognitive outcomes. (Kaiser & Feng, 2015; Schaeffer et al., 2020). Relatedly, the majority of cognitive batteries conducted in rodents are tests of spatial memory, taking advantage of the natural exploratory behaviors of rodents, which may insufficiently model emerging cognitive deficits in human patients and do not capture the behavioral changes, such as mood alterations (Babulal et al., 2016; Robins Wahlin & Byrne, 2011) and increased impulsivity (Bateman et al., 2020; Bidzan et al., 2012; Rochat et al., 2013), which are noted to precede cognitive symptoms. Moreover, tests that include stressors, such as shock (e.g., fear conditioning) or swimming tasks, are not deployed in human clinical trials for aging-related disorders; thus, their utility for translational studies is limited (Silverman et al., 2020). Age is the primary risk factor for developing aging-related disorders, including dementia and neurodegenerative diseases, which include cognitive impairment as a key diagnostic criteria. Thus, it is critical to use animal models that have a lifespan conducive to studying the lengthy process of aging and that have higher-order cognitive processes that better align with that of humans.

To this end, nonhuman primates (NHPs) are important models for studying aging and aging-related disorders as well as cognitive function. NHPs have conserved anatomical and functional organization of the primate brain, which facilitates the study of complex cognitive function well beyond that which can be assessed in rodents (Balsters et al., 2020; Bernardi & Salzman, 2019; Herculano-Houzel, 2009). Across the breadth of laboratory NHP species, there has been a growing interest in the common marmoset (*Callithrix jacchus*) as an improved translational model system for aging and age-related disorders (Perez-Cruz & de Dios Rodriguez-Callejas, 2023; Ross & Salmon, 2019). Compared with other laboratory NHP species that have been studied for aging and aging-related disorders, common marmosets have a relatively short lifespan of up to 12–16 years in captivity (Abbott et al., 2003; Nishijima et al., 2012; Schultz-Darken et al., 2016), providing an opportunistic advantage for studying the full spectrum of aging changes and longevity, including long prodromal periods that precede cognitive decline, in a timeframe that is conducive for research programs. Importantly, similar to other NHPs, marmosets manifest age-related changes across a spectrum of functional and physiological parameters that mirror those in aging humans and with observations of these features reported beginning at ~7–8 years of age (Glavis-Bloom et al., 2022; Nishijima et al., 2012; Perez-Cruz & de Dios Rodriguez-Callejas, 2023; Salmon, 2016; Tardif et al., 2011). For example, similar to aging-related changes in humans, marmosets demonstrate age-related motor, fine motor, and sensorimotor impairments (Workman et al., 2019; reviewed in Murai & Sukoff Rizzo, 2020), decreased hippocampal neurogenesis (Amrein et al., 2015; Charvet & Finlay, 2018), and pathological features that are prevalent in human aging, including sporadic presentation of beta-amyloid (Aβ) plaques in the brain, which is not a natural phenomenon in rodents (Sukoff Rizzo et al., 2023). Marmosets also share several aging-related comorbidities with humans, including diabetes, gastrointestinal disorders, cardiac, and kidney disease, as well as aging-related physical changes in bone density and body composition (Ross et al., 2012; Ross et al., 2017, 2019; Tardif et al., 2011). Notably, the psychological and neurobiological bases of cognitive and affective behaviors have been extensively studied in marmosets and captured in the context of the Research Domain Criterion framework (RDoc) (Oikonomidis et.al., 2017; French, 2019), revealing important neurobiological substrates of neuropsychiatric symptoms, which are prevalent comorbidities in aging-related disorders. Relatedly, several labs have made seminal contributions to studying aging-related changes in cognitive function in marmosets and also have developed robust protocols for specific cognitive tasks across several domains, including learning, memory, and attention (Glavis-Bloom et al., 2022; Rothwell et al., 2021; Sadoun et al., 2019).

Given the success in performance of marmosets on tasks that depend upon a broad range of age-sensitive, cognitive domains, there is an opportunity to develop a battery of individual tasks that capture a spectrum of cognitive functions similar to those measured in the clinic. For example, in humans it is now well established that aging-related mild cognitive impairment and prodromal Alzheimer’s disease (AD) can be detected by using the Cambridge Neuropsychological Test Automated Battery (CANTAB) of visual and spatial memory tests, including delayed matching to sample (Campos-Magdaleno et al., 2021; Juncos-Rabadán et al., 2014), pattern recognition memory (Campos-Magdaleno et al., 2021; Juncos-Rabadán et al., 2014), spatial span (Campos-Magdaleno et al., 2021; Juncos-Rabadán et al., 2014), and paired associates learning (Campos-Magdaleno et al., 2021; Égerházi et al., 2007; Juncos-Rabadán et al., 2014; Junkkila et al., 2012). Indeed, the entire suite of tests for motor skills, executive function, episodic and visual memory, and sustained attention is capable of discriminating MCI from AD (Égerházi et al., 2007; Junkkila et al., 2012) and is correlated with long-term outcome of AD (Abbott et al., 2019; Campos-Magdaleno et al., 2021; Cormack et al., 2015; Fowler et al., 2002). Importantly, the success of CANTAB in humans for determining a baseline of cognitive/behavioral functioning as well as predicting disease outcomes is largely due to the forward and back translation between humans and other animals that underpinned its development (Fray et al., 1996; Weed et al., 1999). Moreover, the NHP CANTAB developed by Robbins and Roberts in 1996 and the more recent application of rodent touchscreen paradigms established by Bussey and Saksida is conducive to closing the translational gap between preclinical disease models and human clinical trials (Barnett et al., 2016; Palmer et al., 2021).

The establishment of a battery of clinically relevant tasks that can be delivered longitudinally with robust test-retest integrity remains an important component of cross-species research in the field of aging and aging-related disorders. Assessing cognitive function throughout the lifespan of marmosets, including adolescence and adulthood is feasible within an 8–10-year research program and should provide important and parallel insights into human aging processes. More specifically, there is an opportunity to capture changes across the lifespan of the sophisticated behavioral repertoire of marmosets that are analogous to many human social and emotional behaviors. Moreover, there is an opportunity to define a dementia-like phenotype in marmosets that could be used to study aging-related disorders that lead to dementia, such as Alzheimer’s disease. This can only be achieved, however, by understanding the variation in the characteristics of normal healthy aging and deciphering those features that diverge and are associated with pathological aging in marmosets (e.g., Aβ deposition), through the application of a wide array of cognitive and behavioral tasks aimed at capturing the spectrum of aging pathology apparent in humans. We propose that the same modality of visual cues and touch used in humans performing touchscreen-based cognitive tasks, such as CANTAB, can be readily delivered to marmosets longitudinally, thereby improving the translation of cognitive function from animal models to humans. Importantly, we have aimed to establish a testing battery with noninvasive measures and in nonrestrained animals, with the goal to develop simple tests that can be used to inform pathological aging relative to chronological age and that could be readily transferrable across laboratories.

The purpose of the present studies was threefold: 1) to identify specific tasks that can be readily trained in marmosets across age and sex, with analogous protocols and outcome measures to human CANTAB, and be incorporated into a testing battery that can be deployed longitudinally throughout the marmoset lifespan; 2) to evaluate the ability of individual marmosets representative across ages and sexes, to readily switch task domains across a variety of different tests delivered consecutively over a broad battery with predefined advancement criteria; and 3) to use the information gained from the present studies to inform an optimal longitudinal testing battery that will be used for future longitudinal studies of aging throughout the lifespan of marmosets with genetic risk for AD. Therefore a priori, there were no preplanned group analysis (e.g., young vs. aged, males vs. females, etc.) but rather presentation of individual animal’s performance across the tasks to demonstrate natural variation within the population. For these studies, the selection of the specific tests for the battery was based on those that capture the spectrum of cognitive functions in humans that are sensitive to aging-related changes (Fernanda et al., 2015; Junkkila et al., 2012; Robbins et al., 1994, 1998). Based on the success of marmosets at performing touchscreen based tasks (Nakamura et al., 2018; Oikonomidis et al., 2017; Spinelli et al., 2004), including those reported to be sensitive to aging (Rothwell et al., 2022; Spinelli et al., 2004; Workman et al., 2019), we designed a testing battery that incorporated the following tasks: progressive ratio (PR) to assess motivation, delayed matching to position (DMTP) to measure spatial working memory, delayed nonmatching to position (DNMTP) to measure cognitive flexibility, Delayed Matching to sample (DMTS) to assess recognition memory, and Serial Reaction Time Task (SRTT) to assess attention. Data presented herein provide insight into initial training and baseline testing across individual subjects, as well as challenges and opportunities of the testing battery for future longitudinal studies. Similar to clinical studies in humans, establishment of baseline performance that can be tracked within-subject throughout lifespan, and integrated with genomic, physiological, behavioral, and pathological data, is the ultimate goal of such a testing battery, which we believe will have broad application not only for AD and aging-related studies but also for other disease domains.

## Materials and methods

### Animals

All animal experimental procedures were performed in accordance with The University of Pittsburgh Institutional Animal Care and Use Committee (IACUC) regulatory policies and were in line with and strictly adhered to the Guide for the Care and Use of Laboratory Animals (National Research Council, 2011). Experimentally naïve, laboratory-bred male and female common marmosets (*Callithrix jacchus,* aged 7 months to 11 years at the beginning of the initial training) were enrolled for these studies. Demographics for individual subjects are provided in Table 1. Subjects were housed in an AAALAC accredited facility at the University of Pittsburgh and maintained at a temperature range of 76–78 °F and 30–70% humidity, with a 12 h:12 h light/dark cycle (lights on at 7 am). Subjects were fed a diet consisting of twice daily provisions of commercial chow including purified diet and supplemented with fresh fruit and vegetables daily with drinking water provided ad libitum. Foraging materials and enrichment also were provided daily.

**Table 1:** Demographics of marmosets enrolled in cognitive training

Subject #	Age (Months)	Sex	Touch Training	PR	DMTP	DNMTP	DMTS	TU-DMTS	4C-SRTT
11	129	M	√ a	√d, X	√ b	√ c, X	-	-	-
12	127	M	√ a	√d	√ b	√ c	-	-	-
24	115	M	√ a	-	-	-	-	-	-
155	112	M	√ a	-	√ b	√ c	-	-	-
17	111	F	√ a	√ b, √d	√ c	-	-	-	-
13	109	F	√ a	-	√ b	√ c	-	-	-
16	108	M	√ a	√ b, √d	√ c, √e	-	-	-	-
20	107	M	√ a	√ b, √d	√ c	-	-	-	-
157	105	F	√ a	-	√ b	-	-	-	-
27	103	F	√ a	√ b, √d	√ c, √e	-	-	-	-
14	101	F	√ a	√d, √e	√ b	√ c	-	-	-
32	99	M	√ a	√ b, √d	√ c, √e	-	-	-	-
51	99	F	√ a	√ c	√ b	-	-	-	-
54	98	M	√ a	-	-	-	-	-	-
49	95	F	√ a	√ b, √d	√ c	-	-	-	-
45	91	M	√ a	√ b, √d	√ c, √e	-	-	-	-
46	89	M	√ a	√ b, √d	√ c, √e	-	-	-	-
38	89	M	√ a, X	-	-	-	-	-	-
21	86	F	√ a	√d	√ b	√ c, X	√e	√f, X	√g
168	83	M	√ a	-	√ b	-	-	-	-
55	82	M	√ a	√ b, √d	√ c, √e	-	-	-	-
58	76	M	√ a	√ b, √c	-	-	-	-	-
123	36	M	√ a	√ b, √d	√ c, √e	-	-	-	-
127	20	M	√ a	-	-	-	-	-	-
9	17	M	√ a	-	√ b	√ c	-	-	-
3	15	M	√ a	√ c	√ b	√d	-	-	-
2	14	M	√ a	√d	√ b	√ c	√e	√f	√g
8	14	F	√ a	-	√ b	√ c	-	-	-
101	12	F	√ a	-	√ b	√ c	-	-	-
218	10	M	√ a	√ b	-	-	-	-	-
275	9	M	√ a	-	-	-	-	-	-
274	9	F	√ a		-	-	-	-	-
286	8	M	√ a	-	-	-	-	-	-
287	7	M	√ a	-	-	-	-	-	-

Subjects are assigned an anonymized subject #. Age (months) indicates start of training. A check mark indicates the subject trained in that specific task. Letters a–g indicate order of testing across tasks for the individual subject, and letter X indicates a failure to meet criteria. Progressive Ratio (PR), Delayed Matching to Position (DMTP), Delayed Non-Matching to Position (DNMTP), Delayed Matching to Sample Training (DMTS), Trial Unique Delayed Matching to Sample (TU-DMTS), Four Choice Serial Reaction Time Task (4C-SRTT). Sex: Male (M), Female (F). Longitudinal retesting for a subject in a specific task is indicated by a second letter within the task column

### Training procedures

**Fig. 1:**
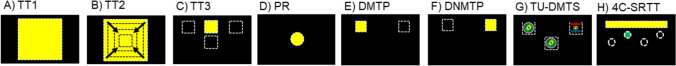
Touch screen tasks in order of progression (**A** through **H**) and related stimuli. The touchscreen is a black background with a clear polycarbonate mask positioned directly in front of the touchscreen with openings specifically fabricated to align to the stimuli for each specific task as indicated by the hashed lines in each illustration (artist rendering, not drawn to precise scale). **A**) Touch training 1 (TT1); **B**) Touch training 2 (TT2); **C**) Touch training 3 (TT3); **D**) Progressive Ratio (PR); **E**) Delayed Match to Position (DMTP); **F**) Delayed Non-matching to Position (DNMTP); **G**) Trial-Unique Delayed Match to Sample (TU-DMTS); **H**) Four Choice Serial Reaction Time Task (4C-SRTT). Stimuli shown are representative illustrations of stimuli for each task (refer to detailed methods including for specific stimuli descriptions)

Figure 1 illustrates the touchscreen tasks in the battery and the order of progression across the tests, as well as the stimuli designated for each task.

### Habituation training

A customized in-house fabricated training nestbox (36 × 30 × 25 cm) made of plexiglass, which allowed for free movement without restraint, was mounted to the homecage during touchscreen training and testing sessions. Sessions were typically conducted 5 consecutive days per week with only a single session per day between 8:30 am and 5:30 pm. Marmosets were trained to enter the training box voluntarily by using positive reinforcement of palatable food rewards (i.e., mini-marshmallows, pieces of sponge cake or marshmallow juice formulated in drinking water—20% or 30% solution). Initial interactions with the marmosets included identifying preferred rewards, which can be specific for individuals (Murai & Sukoff Rizzo, 2020). Interestingly, all marmosets demonstrated marshmallow juice as a salient reward. Only one individual marmoset was stationed in the nestbox during each session, and a cover was placed over the sides of the training box to minimize external cues from the colony room during testing. A closed-circuit video camera was used to monitor behavior during sessions. Following testing, the nestbox chamber was sanitized with 70% ethanol between subjects to avoid the influence of sensory cues. All testing was conducted in the housing room with the exception of the 4 Choice Serial Reaction Time task (4C-SRTT). For this task, subjects entered the touchscreen training box, which was then placed into a sound-attenuated, ventilated chamber (Med-Associates) and transported to an isolated testing room away from the colony to minimize external stimuli (e.g., vocalizations), which may confound performance in this task.

### Initial touch training

Once subjects consistently and voluntarily entered the nestbox, touch-screen training sessions were initiated. A commercially available touch-screen apparatus modified for marmosets (Lafayette Instrument, IN, US) measuring 10.4 inches (800 × 600 pixels) with a black screen was attached to the training nestbox. To encourage touching of the screen, small marshmallow pieces were placed directly onto the screen during initial touch training steps (TT1) described below. To facilitate task switching and minimize extraneous screen touches from the marmosets (e.g., leaning on the screen for stability); for each task, a clear polycarbonate mask was placed in front of the screen that allowed the subject to touch only the areas of the screen that were relevant to that specific task (Fig. 1). One training session per day was conducted with a maximum session time of 20 rewards or 20 min to minimize any potential confounds of decreases in performance related to satiety. All touch training steps consisted of a fixed-ratio 1 (FR-1) schedule of reinforcement. Subjects were required to progress through three steps with a priori advancement criteria as follows:

1. Touch Training Step 1 (TT1)

A yellow square (13 × 16 cm) was presented on the center of the screen (Fig. 1A). When a subject touched the stimulus, the yellow square disappeared and 100 μl of juice reward (20% marshmallow juice) was delivered to a sipper tube mounted on the top of the training box, which completed the trial. Following a 5-s intra-trial interval (ITI), the identical stimulus reappeared. There were no pre-programmed consequences for touching the blank screen during the ITI. Advancement criteria for TT1 was set as completion of 20 trials within 20 min for 3 consecutive days. Critically, a closed-circuit camera was used to confirm the touch reward association in that the 20 trials consisted of touches that were not accidental, but for which the immediate response to the touch resulted in immediate movement of the marmoset to the sipper tube to consume the reward. No additional audio cues were used to signal the presentation of the reward with the exception of the noise output generated from the liquid reward pump (60 dB, 80-msec duration).

2) Touch Training Step 2 (TT2)

As illustrated in Fig. 1B, the session began with the presentation of the identical-sized square stimulus as the previous session (13 × 16 cm; TT1). After completion of five trials, the size of the stimulus sequentially decreased throughout the test session (trials 1–5: 13 × 16 cm; trial 6–10: 9 × 12 cm; trials 11–14: 6.5 × 6.5 cm; trials 15–20: 3 × 3 cm), such that the final size of the stimulus was 3 × 3 cm. Advancement criteria was set as completion of 20 trials within 20 min for 2 consecutive days.

3) Touch Training Step 3 (TT3)

In this final touch training step, as illustrated in Fig. 1C, a small, yellow square (3 × 3 cm) was presented in one of four locations. Advancement criteria was set at 20 responses within 10 min for 3 consecutive days.

### Progressive ratio

Progressive ratio (PR) is a test used to determine a subject’s motivation, which is based on the amount of effort the subject will continue to pursue when the effort required to receive the reward is continually increased (Hodos, 1961). For these studies, the protocol used was similar to that reported by Alexander and colleagues (Alexander et al., 2019). PR training began on a FR-1 schedule, with the stimulus set as a yellow circle (3-cm diameter) presented at the center of the screen (Fig. 1D). Daily sessions were set at a maximum duration of 10 minutes or until a 120-s period without responses elapsed. For each subject, during initial FR-1 sessions, an individual advancement criterion was determined on Day 3, based on the total number of responses completed on Day 2 of the schedule. If the number of responses on Day 3 was greater than the number of responses on Day 2, then the subject would advance to an FR-2 training schedule. If the number of responses on Day 3 was less than the responses on Day 2, then the subjects would continue on the FR-1 schedule until they maintained ≥80% of the average on the first three sessions on FR-1 for 3 consecutive days. Upon meeting advancement criterion on the FR-1 schedule, subjects were advanced to an FR-2 schedule of reinforcement (e.g., 2 responses required to receive the reward). Advancement criterion for FR-2 was based on a requirement of ≥80% of their FR-1 responses from their final day on FR-1. Similar criteria were established for subsequent advancement to FR-3, FR-5, and FR-7. Subjects were advanced to a PR schedule based on either 1) completion of 3 consecutive days on the FR-7 schedule (maximum FR training ratio) or 2) failure to meet advancement criteria on any of the subsequent FR ratio schedules as determined by <80% of the previous FR ratio responses over 3 consecutive days. Once subjects advanced to the PR test session, the response-reward requirement was an incremental schedule similar to previous methods (Alexander et al., 2019). Beginning on an FR-1 and with each trial completed, the FR increased by +1 in subsequent trials under the following incremental response ratios: 1, 2, 3, 4, 5, 6, 7, 8, 10, 12, 14, 16, 18, 20, 22, 24, 28, 32, 36, 40, 44, 48, 52…+4. Breakpoint was defined as the number of total responses completed before a 120-s period without responses elapsed. PR data were analyzed for breakpoint by using the average of three consecutive sessions for each individual.

### Delayed matching to position task

**Fig. 2:**
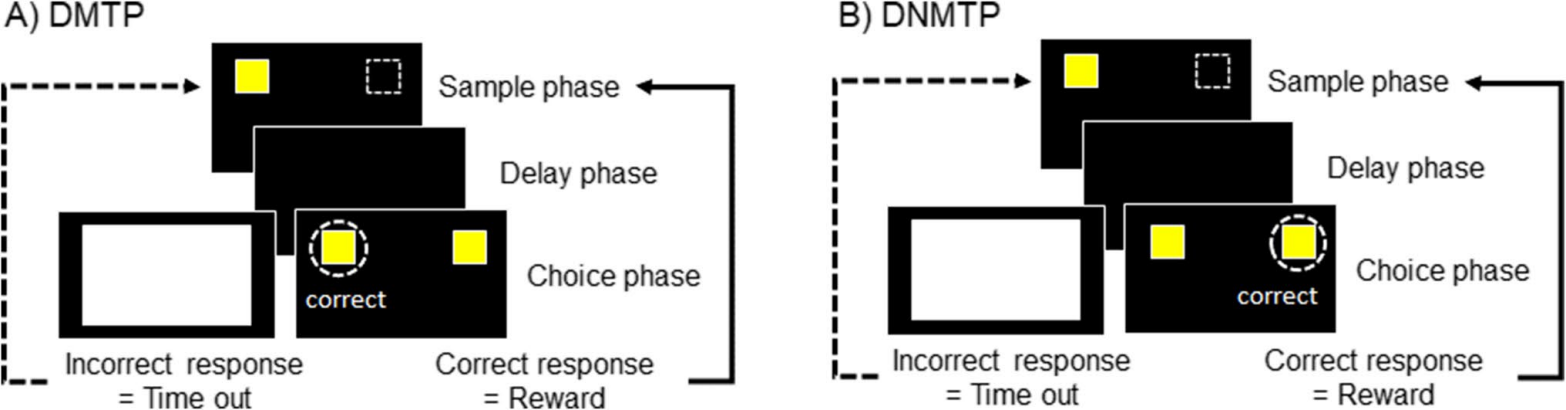
Delayed Match to Position Task (DMTP) and Delayed Non-Matching to Position Task (DNMTP). During the sample phase, the correct stimulus (yellow square) is presented on either left or right sides of the screen. Following the delay phase, a choice phase occurs in which identical stimuli are presented in both positions. During DMTP (**A**), touch responses to the stimulus in the position presented during the sample phase is correct and is rewarded, whereas touch to the opposite position as the sample phase is an incorrect response, which results in a blank white screen for a 5-s timeout (**A**). For DNMTP trials (**B**), during the choice phase, touch responses to the stimulus in the opposite position than the stimulus presented during the sample phase is rewarded

The delayed matching to position (DMTP) is a modification of the methods of Yamazaki et al. (2016) that uses a single visual stimulus throughout the session. DMTP trials consist of two phases: the sample phase and the choice phase (Fig. 2A). During the sample phase, a stimulus (yellow 3- × 3-cm square) is presented in one of two positions (left or right side) with all other areas of the screen blank. The location of the sample is randomized and counterbalanced for left and right positions across trials within each session. The subject must touch the sample to initiate the delay period and subsequent choice phase. During the delay period, the screen is blank and there are no programmed consequences. At the conclusion of the delay period, the identical stimulus is presented in both positions (choice phase) with responses rewarded for a touch to the same location (FR-1) as the sample was presented. Incorrect responses result in a blank white screen for a 5-s time out and no reward. The ITI for both correct and incorrect responses was 5 s. An initial 1-s delay is used for training. As part of establishing this task, we evaluated whether a pretraining phase in which only the correct position is presented during the choice phase would facilitate acquisition. Advancement to the testing component in which both positions are presented during the choice phase requires ≥80% correct for 3 consecutive days and completion of 20 trials within 20 min in the pretraining phase. Data analysis for the testing component (2 choice phase) include number of sessions to criteria (≥80% correct for 3 consecutive days). The % correct for the 3 consecutive days was used to determine advancement criteria. After completion of training on a 1-s delay, the delay period was increased every 3 sessions from 3 s to 12 s (e.g., 3-, 4.5-, 6-, 9-, and 12-s delays). During each subsequent delay assessment, a reduction in accuracy by 20% relative to the individual’s accuracy during the 1-s delay was defined as their delay threshold, and additional delays were not assessed. Once this was determined, subjects were returned to the 1-s delay session until accuracy was consistent with their initial 1-s delay baseline training data (e.g., no statistically significant difference relative to predelay testing).

### Delayed nonmatching to position task

The delayed nonmatching to position (DNMTP) DNMTP task was presented in the subsequent session after the 1-s delay reassessment of DMTP as described above. DNTMP is similar to DMTP except that, during the choice phase, subjects must respond to the opposite position to that which the sample was presented (Fig. 2B). The delay period for DNMTP was fixed at 1 s. A priori criterion for completing DNMTP was ≥80% accuracy for 3 consecutive days. The number of trials to meet the criteria was calculated as the index of cognitive flexibility.

### Trial unique delayed matching to sample (TU-DMTS) task

**Fig. 3:**
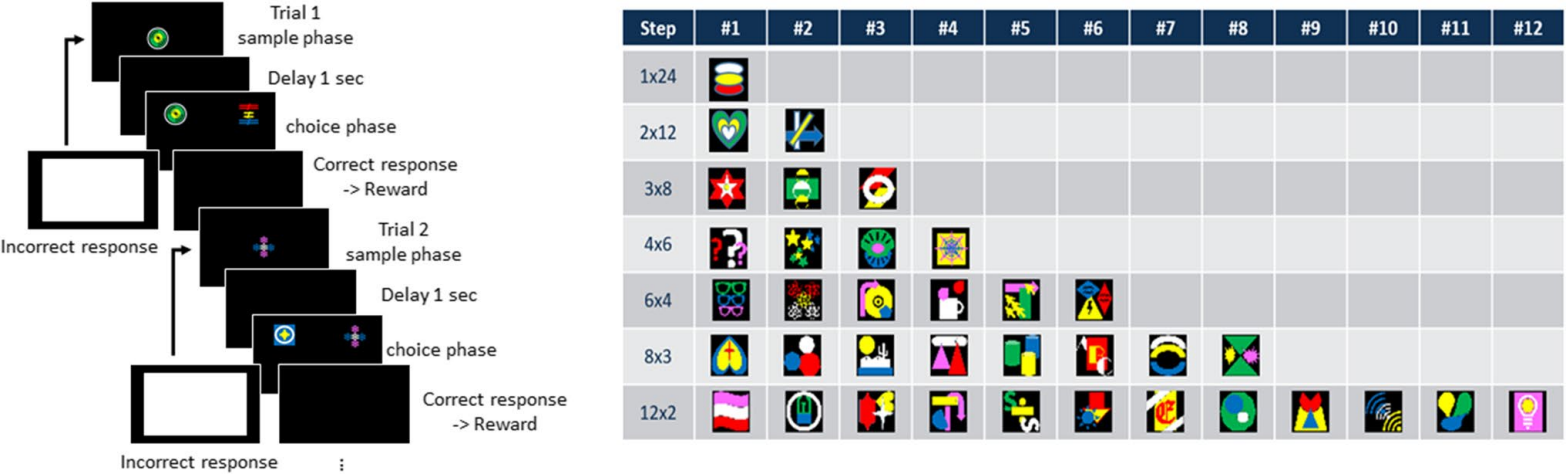
Trial Unique Delayed Matching to Sample Task (TU-DMTS) for the assessment of recognition memory. The TU-DMTS paradigm consisted of a training phase and a testing phase. Unique multicolored stimuli varying across six different colors were used for this task. As illustrated in the left panel, during the sample phase, the subject was required to respond via touch (FR-1) to the sample stimulus presented at the center of the screen. Response to the sample stimulus initiated a 1-s delay period in which the screen was blank and there were no programmed consequences. After the delay, the choice phase was initiated in which two stimuli were presented simultaneously on either left or right sides of the screen. One of the stimuli was identical to the stimulus presented in the choice phase while the other was a novel stimulus. Responses to the correct stimulus were rewarded with liquid reinforcer (20% marshmallow juice) followed by an 8-s ITI. Training for TU-DMTS is initially conducted over the course of 12 training steps. During the initial 12-step training phase, as presented in the table (right panel), a single correct sample stimulus was always presented across the 24 trials (1 × 24) and balanced and randomized across left and right sides. In the second step, two correct sample stimuli were presented across 12 trials each (2 × 12). Subsequent steps introduced an additional correct stimulus (step 3 = 3 × 8 trials each; step 4 = 4 × 6 trials each; step 5 = 6 × 4 trials each, etc.). Once subject met a priori advancement criteria, they were transitioned onto the subsequent steps. Upon completion of all 12 training steps, subjects were advanced to the testing phase in which each of the 24 trials was a novel random stimulus and novel incorrect stimulus

The trial unique delayed matching to sample (TU-DMTS) paradigm consisted of a training phase and a testing phase (Fig. 3). Each session consisted of 24 trials with a maximum session time of 30 min. Unique multicolored stimuli varying across six different colors were used for this task (Fig. 3). Each stimulus consisted of at least three colors with specific attention to avoid a red-green combination because of dichromacy in males and the possibility in females (Abreu et al., 2019; Moreira et al., 2015). During the sample phase, the subject was required to respond via touch (FR-1) to the sample stimulus presented at the center of the screen. Response to the sample stimulus initiated a 1-s delay period in which the screen was blank and there were no programmed consequences. After the delay, the choice phase was initiated in which two stimuli were presented simultaneously on either left or right sides of the screen. One of the stimuli was identical to the stimulus presented in the choice phase; the other was randomly selected from the pool of 50 training stimuli. Responses to the sample stimulus were rewarded with liquid reinforcer (marshmallow juice) followed by an 8-s ITI whilst a response to the alternative choice stimulus resulted in a white screen and no reward followed by an 8-s ITI. Each alternative choice stimulus was presented only once in a session and the order of presentation were randomized in the next training session. A correction trial (identical trial to the previous) was conducted as the subsequent trial for incorrect responses. Correction trials were repeated until the subject touched the correct stimulus. The DMTS training phase, which preceded TU-DMTS and was presented as a step-wise training paradigm, was similar to and an extension of the methods described by Nakamura and colleagues (2019). DMTS training was comprised of 12 steps (Fig. 3). During the initial step, a single correct sample stimulus was always presented across the 24 trials (1 × 24) and balanced and randomized across left and right sides. In the second step, two different sample stimuli were presented across 12 trials each (2 × 12). For the second step and subsequent step, there were two phases: block phase and random phase. During the block phase, one of the sample stimuli was presented for the first 12 trials (#1–12) while the second sample stimulus was presented for trials #13–24. During the random phase, sample stimuli were presented in a randomized order. Accuracy of ≥75% for 3 consecutive days resulted in advanced to the next step-phase session. In the third step, there were three sample stimuli presented across eight trials each (3 × 8) first in the block phase followed by the random phase. During subsequent steps of 4 × 6, 6 × 4, and 12 × 2, an addition phase was included (random block phase), which preceded the random trial phase and was set as presentation of sequential trials of the same sample stimulus but randomized each day as to which trials the block was presented within the test session. For each of the step-phase sessions, advancement criteria was set to ≥75% correct for 3 consecutive days. Novel sample stimuli were introduced in each of the different step sessions and paired with relatively novel, alternative choice stimuli randomly presented from a pool of 50 stimuli. Once subjects advanced through 2 × 12 fixed blocks, random blocks, and random trials, the trial unique (TU) phase of training was introduced. During TU, a novel set of 50 multicolored stimuli were introduced. Combinations of the sample and alternative choice stimuli changed each trial and each stimulus was presented only once in a session. A priori criteria to advance to delay phases required 3 consecutive days of ≥75% correct at the 1-sec delay. Once accuracy was determined for 1-s delay of the TU-DMTS, similar to the DMTP test as described above, longer delays were introduced every 3 sessions (e.g., 3, 4.5, 6, 9, 12, 17 s) until the subject demonstrated >20% reduction in the accuracy (% correct) from the baseline (average of the last 3 sessions in a 1-s delay).

### Four-choice serial reaction time task

**Fig. 4:**
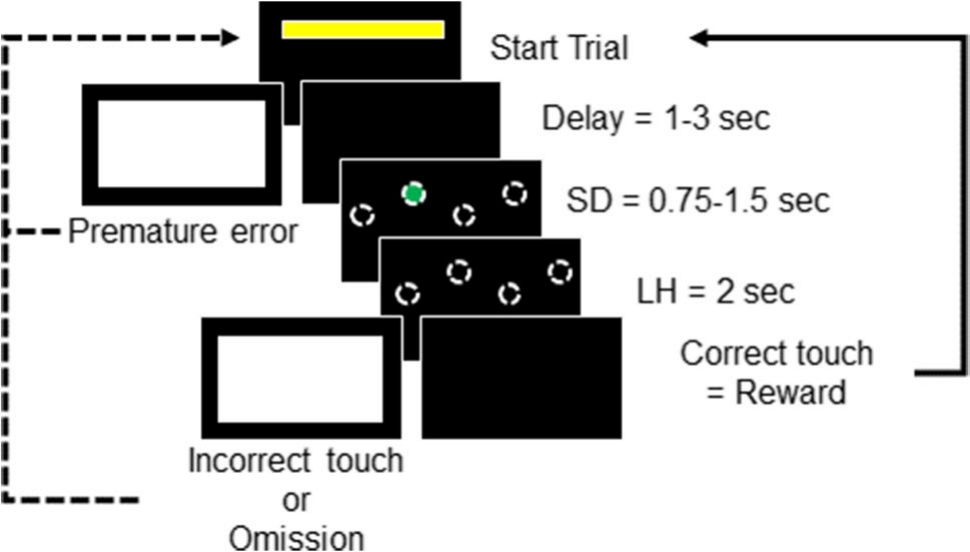
Four-choice Serial Reaction Time Task (4C-SRTT) for the assessment of attention. A trial was initiated when the subject touched the yellow rectangle stimulus at the top of the screen, which resulted in a brief delay phase (1–3 s, randomized) before presentation of the sample phase in which the stimulus (green circle) was randomly presented in one of four locations for a stimulus duration (SD) of 0.75–1.5 s (randomized). Responses during the delay phase were calculated as premature responses resulting in a blank white screen and restart of the trial. Touch responses to the green stimulus in the location it was presented within the limited hold period (LH) rewarded while responses to nontarget areas were calculated as incorrect responses and paired with a blank white screen. Failure to respond during the LH were calculated as omissions

Figure 4 illustrates the four-choice serial reaction time task (4C-SRTT) paradigm, which is a modification of the methods previously described for the 5-choice SRTT in marmosets (Spinelli et al., 2004). The trial was initiated when the subject responded by touching the yellow rectangular bar on the touchscreen.

After a brief delay (1–3 s, randomized), the stimulus (green circle) was presented at one of four locations for a stimulus duration (SD) ranging from 0.75–1.5 s (randomized across trials). Responses during the delay phase were calculated as premature responses, resulting in a blank white screen for a 5-s time-out period followed by restart of the trial. Touch responses to the green stimulus in the location in which it was presented within the limited hold period (LH) were rewarded (100 μL 20% marshmallow juice solution) while responses to nontarget areas were calculated as incorrect responses resulting in a blank white screen for a 5-s time-out period followed by restart of the trial. Failure to respond during the LH were defined as omissions. The session duration was a maximum of 20 min or 30 correct responses, whichever came first. During the initial training phase (Table 2), the delay was set to 0.5 s and the stimulus duration (SD) and the LH were set to unlimited duration. A priori advancement criteria for initial training was completion of 20 correct trials within 20 min with ≥80% accuracy for 2 consecutive days. Once initial training criteria were met, subjects advanced to training sessions that incorporated additional time variables across multiple training steps (Table 2). Once subjects completed the training sessions, testing sessions were conducted that incorporated random presentations of stimulus duration and delays across trials (Table 2). Data were analyzed for accuracy (% correct), premature response errors, omissions, and response latencies.

**Table 2:** Training and testing contingencies for 4C-SRTT in marmosets

	Training Step 1	Training Step 2	Training Step 3	Training Step 4	Training Step 5	Training Step 6	Training Step 7	Training Step 8	Testing Phase
Delay Period (sec)	0.5	0.5, 1	1, 2	1, 2, 3	1, 2, 3	1, 2, 3	1, 2, 3	1, 2, 3	1, 2, 3
Stimulus Duration (sec)	Unlimited	Unlimited	Unlimited	Unlimited	3	2, 3	1.5, 2, 3	1, 1.5, 2	0.75, 1, 1.5
Limited Hold (sec)	Unlimited	Unlimited	Unlimited	Unlimited	6	6	6	6	6
Advancement Criteria	20 correct trials within 20 minutes with ≥80% accuracy for 2 consecutive sessions	30 correct trials within 20 minutes with ≥80% accuracy for 1 session	30 correct trials within 20 minutes with ≥80% accuracy for 1 session	30 correct trials within 20 minutes with ≥80% accuracy for 1 session	≥90% for 1 day	≥90% for 1 day	≥80% for 1 day	≥75% for 3 consecutive days	≥75% for 3 consecutive days

### Statistical analysis

Data are presented for individual subjects as mean ± standard deviation to demonstrate individual variation for specific outcome measures of each task with no preplanned analyses for any specific group (e.g., young vs. aged, males vs. females). Table 1 provides demographic data on the individuals that were able to meet criteria to advance onto specific tests. A priori exclusion criteria were developed as follows: 1) subjects who refused to drink from the sipper tube, which contained the reward; 2) within-subject exclusions for any individual testing session required a documented environmental disruptive issue for that testing day (e.g., veterinary; room maintenance; power outage); or 3) instrument failure (e.g., reward pump not working). The optimal analysis for these studies is to evaluate individual changes within-subject over the course of a lifespan, especially given the genetic and phenotypic heterogeneity of the outbred marmoset population used in these studies, and similar to the studies of aging and cognitive decline in human subjects. Because the present studies were conducted in a cross-sectional cohort with the goal of demonstrating the ability of marmosets to learn a battery of a spectrum of different tasks within a period of time that would allow for longitudinal, annual testing, no group analyses were planned a priori.

## Results

### Touch training

Of the 34 marmosets that were initially enrolled for Touch Training; only one (male, 7 years of age) was excluded because of refusal to drink rewards from the sipper tube despite several sessions to identify a preferable liquid reward. Table 3 presents data for the number of sessions per training step to meet training criteria for 22 male and 11 female marmosets across an age range of 7–129 months. Between 8 to 51 training sessions were required to meet criteria (mean 16.5 days) and confirm successful acquisition of touch-reward associations (Table 3).

**Table 3:** Touch training results for 22 male (M) and 11 female (F) marmosets

Subject Demographics			Touch training Days to meet criteria			
Subject	Sex	Age (months)	Step 1	Step 2	Step 3	Total
11	M	129	3	2	13	18
12	M	127	3	3	8	14
24	M	115	3	45	3	51
155	M	112	3	4	13	20
17	F	111	10	3	3	16
13	F	109	3	2	3	8
16	M	108	3	2	3	8
20	M	107	3	32	4	39
157	F	105	3	2	14	19
27	F	103	4	7	8	19
14	F	101	8	4	11	23
78	M	99	3	25	12	40
51	F	99	3	8	3	14
54	M	98	3	2	4	9
49	F	95	6	3	3	12
45	M	91	3	6	3	12
46	M	89	5	2	3	10
21	F	86	3	5	5	13
168	M	83	3	5	3	11
55	M	82	3	2	3	8
58	M	76	6	8	4	18
123	M	36	4	2	3	9
127	M	20	3	2	5	10
9	M	17	3	2	6	11
3	M	15	3	2	3	8
2	M	14	3	-	5	8
8	F	14	3	-	5	8
101	F	12	3	2	3	8
218	M	10	20	2	3	25
275	M	9	3	2	7	12
274	F	9	3	2	7	12
286	M	8	3	9	3	15
287	M	7	3	27	5	35

### Progressive ratio

**Fig. 5:**
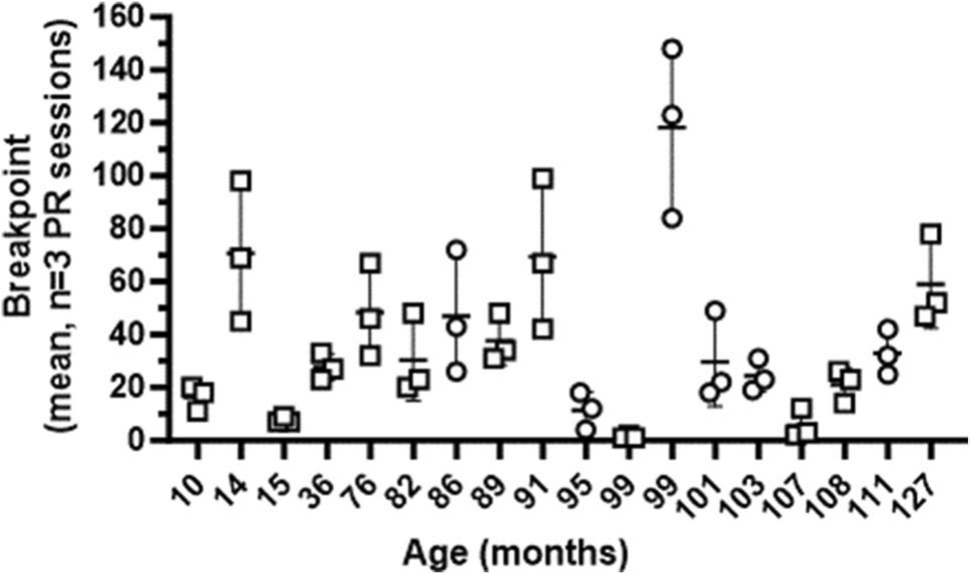
Progressive ratio (PR) breakpoint data for 12 male (M) and six female (F) marmosets across ages. Breakpoints for three consecutive PR testing sessions presented as mean ± standard deviation (STDEV). Circles and squares indicate females and males, respectively

Data for 18 male and female subjects (age range 10–127 months) that completed the PR task are presented in Fig. 5. Breakpoint was defined as the number of total responses completed before a 120-s period elapsed without responses. Longitudinal retest results presented for breakpoint from a single day retest at least 6 months from the initial test Table 4.

**Table 4:** Data for individual marmosets evaluated for assessment of motivation as measured by the progressive ratio (PR) task

		Initial PR		Longitudinal PR	
Subject #	Sex	Age at initial Test (months)	Average Breakpoints ± STDEV	Age at Retest (months)	Breakpoint
12	M	127	59.0 ± 16.64	-	-
17	F	111	33.0 ± 8.54	122	62
16	M	108	21.0 ± 6.24	129	14
20	M	107	5.7 ± 5.51	117	56
27	F	103	24.3 ± 6.11	112	33
14	F	101	29.7 ± 16.86	133	138
32	M	99	1.3 ± 0.58	108	61
51	F	99	118.3 ± 32.25	-	-
49	F	95	11.3 ± 7.02	101	102
45	M	91	69.3 ± 28.57	99	135
46	M	89	37.7 ± 9.07	99	22
21	F	86	47.0 ± 23.26	-	-
55	M	82	30.3 ± 15.37	94	31
58	M	76	48.3 ± 17.62	91	61
123	M	36	27.7 ± 50.3	46	42
3	M	15	7.7 ± 1.15	-	-
2	M	14	70.7 ± 26.54	-	-
218	M	10	16.3 ± 4.73	-	-

Breakpoint was defined as the # of total responses completed before a 120-s period without responses elapsed or 20 min. Data are calculated as the average breakpoint over 3 consecutive sessions for initial test ± STDEV and for the single-day results from a longitudinal retest. Blank entries (–) indicate the subject did not undergo longitudinal retesting. Sex: Male (M), Female (F)

### DMTP

**Fig. 6:**
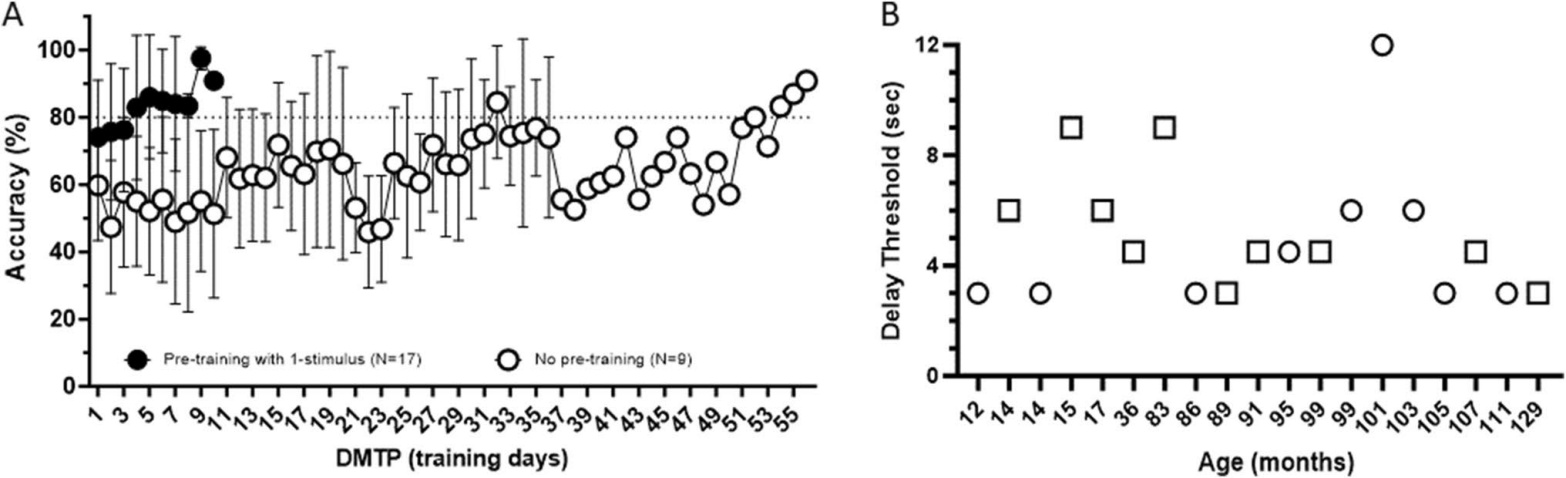
Data from DMTP training and testing in marmosets. **A** Training data. Closed circles indicate data from subjects that initiated pre-training with only the one correct stimulus in the choice phase (N = 17); open circles are data from subjects that did not undergo pretraining (N = 9). Data are presented as mean ±STDEV. **B** Delay threshold of each subject during the testing phase. The delay threshold was defined as the delay period (seconds) in which the subject showed >20% accuracy reduction from the baseline. Circles and squares indicate females and males, respectively

Training data for 15 male and 11 female subjects that completed DMTP are presented in Table 5 and Fig. 6. The average number of training days to meet criteria for subjects that underwent the one-choice pretraining phase was 12.53 ± 4.73 days (single location during the choice phase; n = 17), whereas the number of training days to meet criteria in the subset of subjects trained without the pretraining phase (n = 9) was 24.22 ± 15.11 days, suggesting that the pretraining phase may facilitate acquisition of the DMTP task. During the delay sessions of the testing phase of DMTP, as expected, there was a delay-dependent decrease in accuracy (Table 6). Of the 26 marmosets enrolled in this study, five did not show a 20% reduction in accuracy despite delays of >12 s. Interestingly all five of these marmosets were greater than age 6.5 years. Observation of video recordings indicated a potential learned strategy in that these subjects maintained their position during the delay phase on the same side as the sample; thus, longer delay phases were not undertaken. This observation has been reported by others for this task in NHP species, including marmosets (Roberts et al., 1994; Spinelli et al., 2004; Wong et al., 2023; Yamazaki et al., 2016), and indicate that these subjects have indeed learned the task, although probing of spatial working memory may be limited in this paradigm due to these behaviors. Table 7 provides data on the average response latencies (reaction times) in each delay session for each individual. In general, as the delay period increased, even with subjects that used the waiting strategy, there was an increase in choice latencies for the incorrect stimulus relative to the correct stimulus, which is consistent with previous reports for this task and indicates that subjects are indeed relying on memory (Link, 1982; Wong et al., 2023). During the longitudinal retest of performance at the 1-s delay, which was conducted after all delay sessions were completed, subjects demonstrated comparable accuracy to their previous 1-s delay performance (Table 5).

**Table 5:** Data for individual marmosets of the number of days to meet criteria (>80% accuracy) for the DMTP 1-s delay training

			#of Days to Criteria (initial Test)			Longitudinal Retest	
Subject #	Sex	Age (months)	1-stimulus Pre-training Choice phase	2-stimulus Pre-Training Choice phase	Total	Test-retest period (weeks from previous test)	# of days required for retraining (>80% correct)
11	M	129	4	6	10	-	-
12	M	127	4	6	10	-	-
155	M	112	7	8	15	-	-
17	F	111	-	35	35	-	-
13	F	109	4	5	9	-	-
16	M	108	-	12	12	15	1
20	M	107	-	20	20	-	-
157	F	105	3	5	8	-	-
27	F	103	-	16	16	10	1
14	F	101	4	5	9	-	-
32	M	99	-	18	18	5	1
51	F	99	2	5	7	-	-
49	F	95	5	7	12	-	-
45	M	91	-	15	15	9	1
46	M	89	-	36	36	6	1
21	F	86	4	9	13	-	-
168	M	83	5	5	10	-	-
55	M	82	-	10	10	9	1
123	M	36	-	56	56	8	1
9	M	17	8	6	14	-	-
3	M	15	6	6	12	-	-
2	M	14	6	9	15	-	-
8	F	14	8	6	14	-	-
101	F	12	18	10	28	-	-
274	F	9	8	7	15		-
286	M	8	5	8	13		-

Blank entries (–) indicates that the subject did not undergo the 1-stimulus pretraining or the longitudinal retest. Sex: Male (M), Female (F)

**Table 6:** Accuracy (% correct) for individual marmosets evaluated for assessment of spatial working memory as measured by the DMTP task

			Accuracy (% correct)						Delay Threshold (sec)	Re-Test on 1-sec from previous test	Longitudinal Retest								
Subject #	Sex	Age (months)	1	3	4.5	6	9	12			Test-Retest period (weeks) from previous test	% correct @ 1 sec delay	% correct @ 3 sec delay	correct @ 4.5 sec delay	% correct @ 6 sec delay	% correct @ 9 sec delay	% correct @ 12 sec delay	% correct @ 1 sec delay	Delay Threshold (sec)
11	M	129	90.9	57.8	-	-	-	-	3	100	-	-	-	-	-	-	-	-	-
12	M	127	90.8	92.3	88.2	83.3	88.2	75.9	>12	100	-	-	-	-	-	-	-	-	-
155	M	112	84.5	81.1	70.6	81.1	68.2	67.4	>12	88.7	-	-	-	-	-	-	-	-	-
17	F	111	86	55.6	90.9	-	-	-	3	86.4	-	-	-	-	-	-	-	-	-
13	F	109	97.6	84.5	90.9	81.1	83.3	77.9	>12	98.4	-	-	-	-	-	-	-	-	-
16	M	108	90.1	93.1	90.9	75.3	84.4	73.3	>12	100	15	100	-	-	-	-	-53.8	100	12
20	M	107	98.4	81.5	77	-	-	-	4.5	91	-	-	-	-	-	-	-	-	-
157	F	105	96.8	66.3	-	-	-	-	3	100	-	-	-	-	-	-	-	-	-
27	F	103	83.5	72	68.5	63.2	-	-	6	92.9	10	90.9	-	-	69	-	-	95.2	6
14	F	101	96.8	80	96.8	85.7	82.2	70.6	12	98.4	-	-	-	-	-	-	-	-	-
32	M	99	90.3	75.5	50.7	-	-	-	4.5	100	5	100	-	62.5	-	-	-	95.2	4.5
51	F	99	89	88.9	70.2	67	-	-	6	88.7	-	-	-	-	-	-	-	-	-
49	F	95	87.4	71	55.9	-	-	-	4.5	87	-	-	-	-	-	-	-	-	-
45	M	91	96.8	78.5	78.5	-	-	-	4.5	95.4	9	100	-	-	71.4	-	-	100	6
46	M	89	91	68.5	-	-	-	-	3	100	6	100	66.7	-	-	-	-	100	3
21	F	86	88.2	61.2	-	-	-	-	3	87.3	-	-	-	-	-	-	-	-	-
168	M	83	100	88.2	96.8	90.9	66.7	-	9	96.8	-	-	-	-	-	-	-	-	-
55	M	82	86	69.4	76.3	70.9	83.3	95.2	>12	100	9	100	-	-	-	-	90.5	100	>12
123	M	36	87.1	67.7	62.7	-	-	-	4.5	96.8	8	87	-	46.5	-	-	-	83.3	4.5
9	M	17	97	91.4	91.3	68	-	-	6	100	-	-	-	-	-	-	-	-	-
3	M	15	98.4	90.9	85.5	78.9	65.2	-	9	97.2	-	-	-	-	-	-	-	-	-
2	M	14	88.2	77.9	69.7	65.2	-	-	6	89.8	-	-	-	-	-	-	-	-	-
8	F	14	92.3	63.8	-	-	-	-	3	100	-	-	-	-	-	-	-	-	-
101	F	12	89.8	67.4	-	-	-	-	3	91.3	-	-	-	-	-	-	-	-	-

The delay threshold was the delay (seconds) in which the subject showed ≥20% reduction in accuracy relative to baseline performance during the initial 1 second delay. Longitudinal retesting conducted in a subset of subjects 5–15 weeks after initial testing. Blank entries (–) indicate the subject was not tested under the conditions noted based on delay threshold criterion or not yet evaluated longitudinally. Sex: Male (M), Female (F)

**Table 7:** Choice latency (sec) of individual marmosets evaluated for assessment of spatial working memory as measured by the DMTP task

			1 second delay		3 second delay		4.5 second delay		6 second delay		9 second delay		12 second delay	
Subject #	Sex	Age (months)	Average Correct Latency (sec)	Average incorrect Latency (sec)	Average Correct Latency (sec)	Average incorrect Latency (sec)	Average Correct Latency (sec)	Average incorrect Latency (sec)	Average Correct Latency (sec)	Average incorrect Latency (sec)	Average Correct Latency (sec)	Average incorrect Latency (sec)	Average Correct Latency (sec)	Average incorrect Latency (sec)
11	M	129	3.97	14.3	6.68	6.25	-	-	-	-	-	-	-	-
12	M	127	2.07	2.07	1.96	7.25	2.54	1.98	3.28	2.6	2.55	4.14	3.05	5.59
155	M	112	2.81	16.73	3.83	4.91	3.91	3.4	3.83	6.8	3.49	7.81	4.39	5.16
17	F	111	1.84	2.21	6.89	3.13	-	-	-	-	-	-	-	-
13	F	109	1.83	1.43	2.4	3.65	1.81	2.94	3.69	11.48	1.85	3.45	3.54	4.88
16	M	108	2.19	4.03	1.62	7.35	5.28	15.53	7.94	54.85	27.85	177.51	13.91	16.15
20	M	107	2.33	3.29	4.29	16.47	5.2	6.35	-	-	-	-	-	-
157	F	105	1.28	6.21	5.32	9.47	-	-	-	-	-	-	-	-
27	F	103	1.82	8.43	7.75	13.44	16.48	12.27	4.2	7.4	-	-	-	-
14	F	101	1.53	3.27	3.97	4.25	2.8	10.99	2.2	9.87	3.18	6.07	2.83	3.99
32	M	99	1.66	1.95	7.18	10.28	12.09	8.73	-	-	-	-	-	-
51	F	99	3.18	3.99	1.77	2.95	3.55	4.95	2.6	4.25	-	-	-	-
49	F	95	3.59	9.53	6.54	14.82	13.46	7.62	-	-	-	-	-	-
45	M	91	1.83	10.84	3.51	4.45	5.34	18.7	-	-	-	-	-	-
46	M	89	2.9	15.04	2.41	17.91	-	-	-	-	-	-	-	-
21	F	86	1.61	2.05	4.93	5.19	-	-	-	-	-	-	-	-
168	M	83	1.59	-	1.97	3.33	1.84	2.91	2.2	4.09	3.05	3.58	-	-
55	M	82	5.61	6.4	3.55	5.59	1.9	6.13	7.48	26.23	18.05	87.54	1.85	4.19
123	M	36	1.74	2.78	4.02	3.78	289	4.47	-	-	-	-	-	-
9	M	17	2.06	1.49	1.41	8.7	1.99	12.18	3.5	4.51	-	-	-	-
3	M	15	1.94	26.58	2.4	2.18	7.77	2401	9.78	22.31	9.04	11.68	-	-
2	M	14	3.72	18.57	1.67	2.24	4.05	19.6	3.52	11.12	-	-	-	-
8	F	14	1.09	2.62	1.2	1.92	-	-	-	-	-	-	-	-
101	F	12	2.23	2.32	2.66	6.26	-	-	-	-	-	-	-	-

Blank entries (–) indicate the subject was not tested under the conditions noted based on delay threshold criterion. Sex: Male (M), Female (F)

### DNMTP

**Fig. 7:**
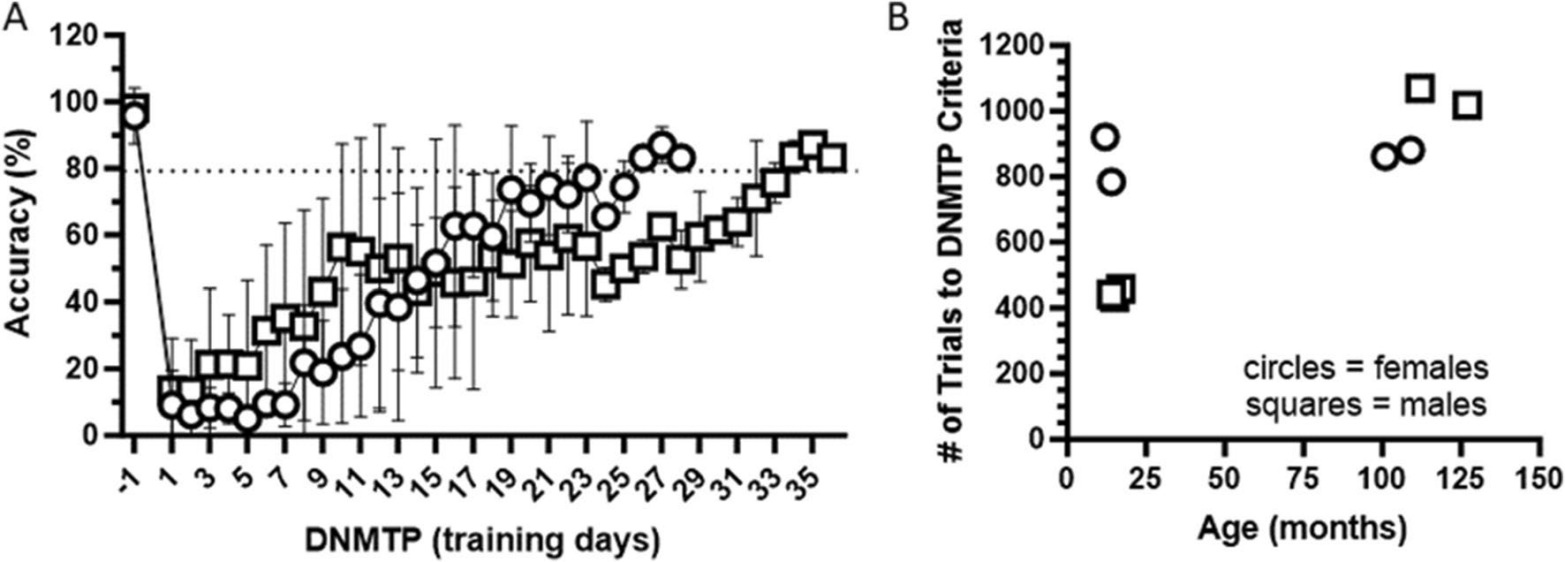
Acquisition of DNMTP after completion of DMTP task. **A** As expected, relative to the final day of DMTP testing (Day −1), which preceded DNMTP training, performance was reduced to <20% correct responses (accuracy) on Day 1. For illustration purposes, average % correct for males (n = 5; square symbols) and average % correct for females (n = 4 circle symbols) over the course of training days is presented (±STDEV). **B** Trials to completion criteria for DNMTP for individual marmosets. Circles and squares indicate females and males, respectively

As illustrated in Fig. 7, on the first day of DNMTP as expected, accuracy was reduced by >80% relative to within-subject performance on DMTP in all subjects. The number of trials to meet criteria for DNMTP for each individual marmoset (≥80% correct for 3 consecutive days) is presented in Table 7. Two animals failed to meet a priori criteria. Subject #21 was trained for 1545 trials but did not improve accuracy above 80% correct. Subject #11 stopped touching the stimulus as the training progressed, so the training was suspended at 871 trials and training was discontinued Table 8.

**Table 8:** Data for individual marmosets evaluated for assessment of cognitive flexibility as measured by the DNMTP task

Subject #	Sex	Age (months)	#of Trials to Criteria
11	M	129	871 (X)
12	M	127	1017
155	M	112	1069
13	F	109	881
14	F	101	861
21	F	86	1545 (X)
9	M	17	458
3	M	15	432
2	M	14	441
8	F	14	784
101	F	12	921

Data are presented as # of total trials required to meet a priori criteria of ≥80 % for 3 consecutive testing days. Two marmosets, indicated with “X”, did not meet the criteria. Sex: Male (M), Female (F)

### Delayed matching to sample

**Fig. 8:**
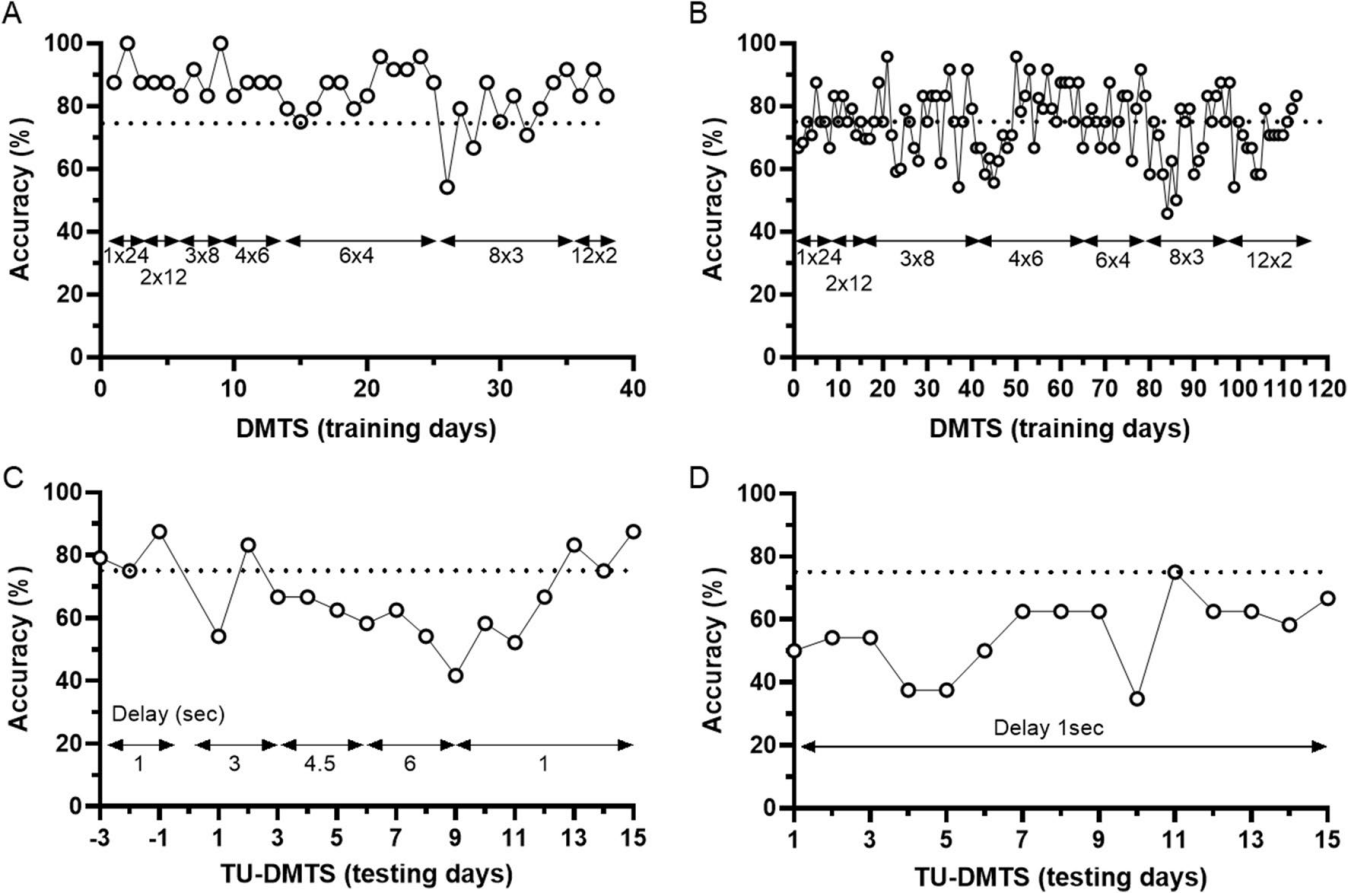
Performance of individual marmosets in Delayed Matching to Sample training and Trial Unique Delayed Matching to Sample Testing (TU-DMTS) for recognition memory. During the training phase, novel stimuli are introduced in sequential sessions as performance meets predetermined advancement criteria (1 × 24 = 1 novel sample stimulus for 24 trials; 2 × 12 = 2 novel sample stimuli across 12 trials each; 3 × 8 = 3 novel sample stimuli across 8 trials each, etc.). Training data from a 1-year-old male (**A**) and a 7-year-old female (**B**). Once subjects complete DMTS training, during the testing phase unique stimuli are presented during every trial within each test session. TU-DMTS testing data for 1-year-old male (**C**) and 7-year-old female (**D**)

Data from the DMTS training and TU-DMTS testing phases are presented in Fig. 8. Both subjects (Subject #2, male, aged 1 year; Fig. 8A; and Subject #21, female, aged 7 years; Fig. 8B) met the training criteria and advanced to TU-DMTS testing. During the Trial Unique presentations, Subject #2 demonstrated delay-dependent reductions in accuracy with a >20% reduction observed at the 6-s delay (Fig. 8C). Subject #21 failed to exceed >75% accuracy over 18 days during Trial Unique-DMTS during the 1-s delay period (on average 54.6%) and was not advanced to longer delays (Fig. 8D).

### Four-choice serial reaction time task

**Fig. 9:**
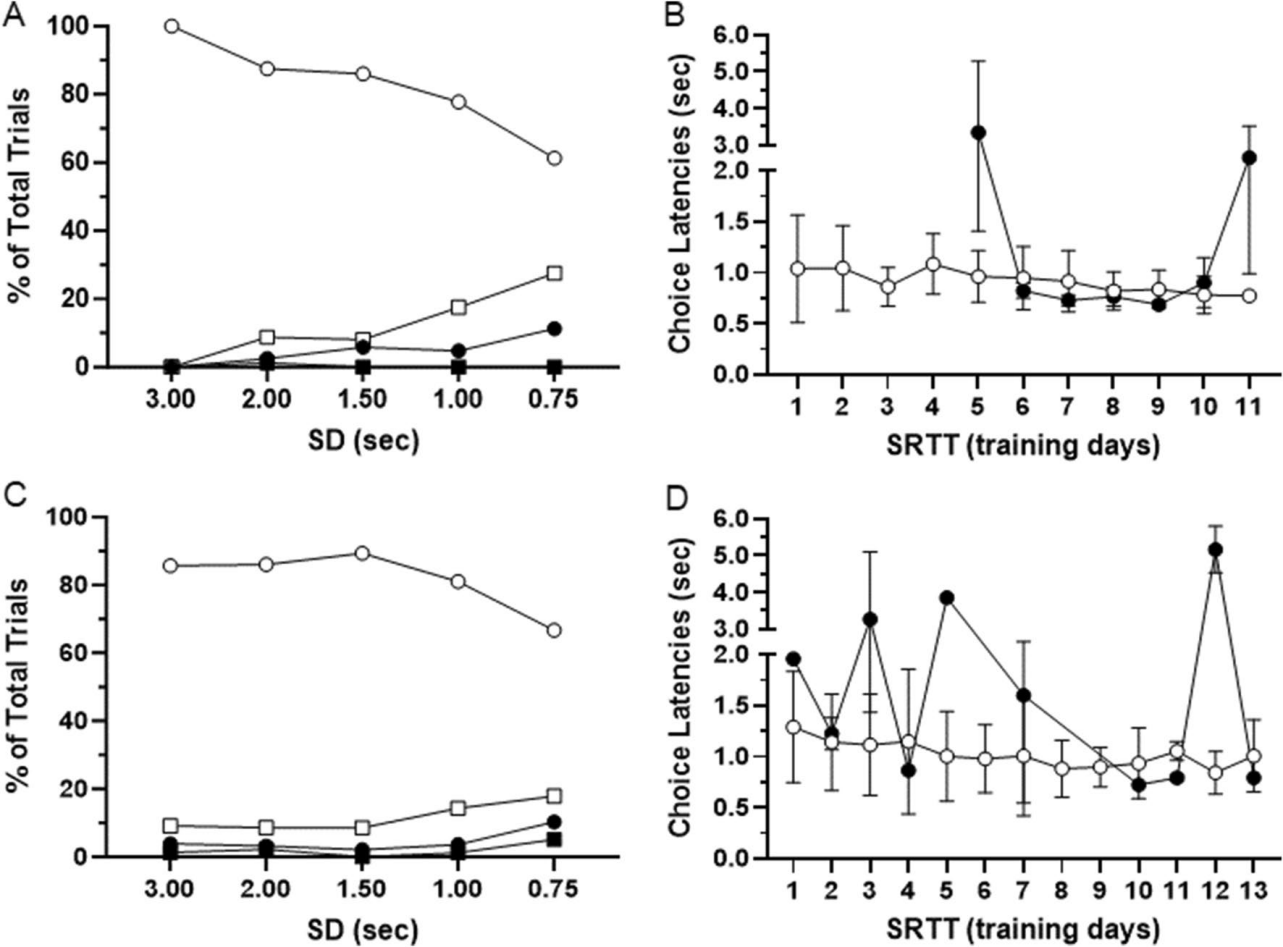
Four Choice Serial Reaction Time Task (4C-SRTT) in marmosets. As expected, subjects demonstrate stimulus duration (SD) dependent reductions in accuracy (% correct responses, open circles; % incorrect responses, closed circles) with increased response latencies during incorrect responses. Omissions are represented by open squares and premature responses by closed squares. **A–B** Response choices and choice latencies, respectively for subject #2. **C–D** Response choices and choice latencies, respectively for subject #21. Data are presented as mean ± STDEV

To assess attention, subjects that completed TU-DMTS testing were advanced to the 4C-SRTT. During this task, subjects are trained in a testing room isolated from the colony to minimize distractions and interference from external stimuli. Both subjects (Subject #2, 1-year-old male, and Subject #21, 7-year-old female) completed the training steps in 16 sessions. As expected, (Fig. 9), subjects demonstrate stimulus duration (SD)-dependent reductions in accuracy (% correct responses) with increased and inconsistent choice latencies during incorrect responses. Overall, omissions were more common than incorrect responses.

## Discussion

The results from the current study support the successful development of a comprehensive within-subject cognitive testing battery in marmosets using the same modality of visual stimuli and touch response as in humans to capture a spectrum of cognitive domains inclusive of attention, spatial working memory, behavioral flexibility, motivation, and recognition memory, and in which marmosets can readily be trained using positive reinforcement. Marmosets across ages were readily capable of switching across tasks. They demonstrated robust motivation to perform an array of different tasks for a salient and preferred reward (marshmallow juice) in the absence of requiring any food or water restriction. The latter is critical, because it would be challenging for longitudinal experiments to depend upon food and/or water restriction over the lifespan of the test subject. We believe that this battery approach provides insight into behavioral performance across a spectrum of cognitive domains beyond that which can be evaluated by using a single test, thereby maximizing the value of these subjects in studies of aging and aging-related disorders. In addition to the utility of a longitudinal and comprehensive testing battery for aging studies, it is essential to measure an extensive repertoire of behaviors to determine how the influence of a specific cognitive domain (e.g., attention) may explain or confound the outcome of another domain (e.g., spatial working memory). This approach is already being used as part of a multi-institutional consortium focused on the generation, characterization, and validation of Marmosets As Research Models of Alzheimer’s Disease (MARMO-AD) (Sukoff Rizzo et al., 2023), described in detail below.

Before establishing this testing battery for MARMO-AD, the present studies were initially and specifically planned to evaluate variation across a diverse population of typical laboratory marmosets, under the specific environmental conditions in our colony, and to understand the ability of marmosets to readily switch tasks across cognitive domains. We enrolled 23 male and 11 female marmosets that spanned an age range of 7 months through 11 years. Despite different rates of acquisition across tasks, which was expected, the data from these studies demonstrate the ability of marmosets to advance from one task to another over sequential sessions, which is in line with previous reports for within-subject cognitive testing batteries in marmosets (Spinelli et al., 2004). Our testing battery has many similarities to Spinelli and colleagues, including a within-subject testing battery, similar methods, and intrasession results for assessing PR, and the inclusion of a 5-choice serial reaction time test (SRTT), which inspired our SRTT approach (Spinelli et al., 2004). Notably, Spinelli and colleagues employed the WGTA instrument and trained marmosets to use the lever to initiate the SRTT trials, while our touchscreen apparatus does not have the option for a lever (Spinelli et al., 2004). As a modification to the task, we designed the trial initiation by touch to a yellow rectangle at the top of the screen and minimized potential for center-touch bias by providing only a 4-choice scenario without a center fifth choice. Our data for demonstrating the expected stimulus-dependent reductions in accuracy are in line with the validation data for 5-choice SRTT in marmosets and indicate cross-laboratory replicability for assessing attention in marmosets in SRTT tasks as well as confirming cross-species translatability (Spinelli et al., 2004). Other major differences of our testing battery to that of other published marmoset cognitive batteries beyond testing order and selection of specific tests include the use of a single visual stimulus (yellow square) for all DMTP and DNMTP testing to ensure results are exclusive to spatial location and not influenced by stimulus, as well as pretraining for DMTP by using a one-stimulus location paradigm, which facilitated the ability to acquire the two-stimulus choice task. Moreover, we developed an extensive step-wise training for DMTS to evaluate recognition memory, including the more challenging TU-DMTS paradigm. Our extensive step-wise training on DMTS builds on the work of Nakamura and colleagues, which was developed to overcome limitations reported for marmosets in the ability to perform the CANTAB DMTS tasks (Nakamura et al., 2018; Ridley et al., 1988; Spinelli et al., 2004). Using this step-wise training paradigm, we describe in detail in the present manuscript and delivered to the two subjects that successfully completed the other preceding tests in the battery, both were successful in learning DMTS, which is consistent with the high success rate reported by Nakamura et al. (2018). Interestingly, despite similar performance on DMTS by both subjects, the 10-year-old subject failed to perform above chance levels on the TU-DMTS. Although more data from additional subjects will be necessary to make definitive conclusions and to date several additional marmosets are currently undergoing DMTS training, it may indicate that TU-DMTS may be more sensitive to probing recognition memory and implicates the importance of this paradigm for longitudinal aging studies despite the initial lengthy investment in training.

To date, we have not attempted to switch tasks within the same testing day, as we also are cognizant of satiety factors that may confound performance in longer test sessions; hence, our protocols are limited to no longer than 30 min and no more than 30 rewards per testing session with only a single testing session per day. While at the time of this submission not all subjects advanced through all tasks, which is related to individual variation in task acquisition as presented herein, studies in this population are ongoing and following the predetermined advancement criteria described in the *Methods* and the *Results*, as well as planned longitudinal studies in subsequent years. It is the goals of MARMO-AD to ultimately phenotype more than 200 marmosets in our colony across their lifespan, estimating up to 12 years of longitudinal testing. Indeed, the initial investment to train the marmosets for these tasks is intensive and was planned to require 6 months of initial training, which is subject-dependent and with greater time required to meet criteria for some tasks relative to others. However, many of the steps required as part of the initial training are not required for subsequent testing. Thus, the time required to complete the longitudinal testing each year throughout the lifespan is vastly reduced to 8–12 weeks per year. Already we have observed a significant reduction in reacquisition for tasks in which subjects have been retested, including only a single day retest requirement for PR (Table 4) and a single day retrain requirement for DMTP (Table 5), suggesting that the proposed longitudinal design will be capable of capturing initial rates of learning (as measured by days/trials to criterion), peak and plateau performance during retesting, and an eventual cognitive decline. This design also addresses a strength of the marmoset model in its ability to recall multiple sets of cognitive testing rules across significant periods of time.

An important component of these studies within the context of establishing the battery was the order of testing of the different tasks. Once subjects acquired touch training and pair-reward associations, we initially advanced to progressive ratio as the next test in the battery to assess motivation early on in the training process. Variability in motivation in performance and task acquisition is a strong consideration in developing a cognitive-behavioral pipeline (Spinelli et al., 2004). Of note was our discovery of the salience of liquid marshmallow solutions as a reward, which was inspired by anecdotal reports from marmoset researchers on marmosets’ preference for marshmallows. All but one of the subjects was receptive to the marshmallow juice reward, suggesting there may be a near-ubiquitous preference for marshmallow juice among common marmosets being trained in operant-based tasks, including touchscreen cognitive tests. Given the long-term longitudinal nature of our studies, identifying a salient reward that is robust enough without the need for water or food restriction was critical to the success of our testing paradigm, although it has been previously reported that task acquisition can be facilitated with food and/or water restriction (Spinelli et al., 2004). While the low rate of subject attrition due to unwillingness to perform or consume the marshmallow juice reward was encouraging, we observed lower performance than expected in several marmosets. Indeed, alterations in motivation are a component of aging and aging-related disorders, such as AD, and while there was no clear distribution of high and low performers related to subject age in the present studies, the natural variation in performance of marmosets is not unexpected and provides insight into the importance of within-subject longitudinal testing evaluating relative changes from baseline as an important factor. While we used standardized training protocols that slowly introduced incremental increases in the FR before PR (Alexander et al., 2019), in the lower performing subjects, there was some concern of potential extinction at the higher ratios (e.g., FR-7). Along with the lengthy training steps for this task, we were prompted to move the PR task to the end of the testing battery for future studies as well as for longitudinal testing. Interestingly, longitudinal retesting for PR in a subset of individuals at the conclusion of the testing battery revealed consistent breakpoints relative to initial testing even after several months of an inter-testing interval. This indicates that motivation in this assay as measured by breakpoint may be reflective of trait rather than state. Additional longitudinal retesting with aging will be important to further understand these findings, especially with respect to aging, pathology, and genetics, including identifying quantitative trait loci through sequencing related to this behavior, which are planned through the MARMO-AD consortium (Sukoff Rizzo et al., 2023). Another important observation in these studies was that some marmosets during the delay component of the DMTP task adopted a strategy of waiting in front of the sample stimulus. This type of strategy has been reported previously in marmosets (Roberts et al., 1994; Spinelli et al., 2004; Yamazaki et al., 2016), although other reports suggest this not to be a confound (Wong et al., 2023.). Relatedly, behavioral strategy also may be an aging-related variable. Sadoun et al. (2019) reported that integrating positive/negative feedback deficits appeared in marmosets as early as age four. However, given the concern that in these subjects working memory capacity in this task was not being tested as intended, based on these data from the present studies, future DMTP studies will be trained and tested with only a 1-s delay to the predetermined criteria, then advanced to DNMTP to assess cognitive flexibility (days to >80% criteria), followed by testing of delays as part of DNMTP with a similar incremental delay testing paradigm as described for DMTP. While it is possible that a similar waiting strategy could be learned in the DNMTP, an alternative solution may be to place the sipper tube for the reward at the back of the apparatus. Regardless, based on the analysis of response latencies for both correct and incorrect choices, despite the waiting strategy, the increase in latencies observed during the incorrect responses are likely to reflect working memory (Link, 1982; Wong et al., 2023). Furthermore, the planned longitudinal testing of this paradigm within subject may reveal alterations in behavioral strategies over time and provide an index of cognitive decline in this domain. Another observation from the DMTP paradigm was the use of the pretraining one stimulus choice phase to facilitate training. This was introduced after observing the lower-than-expected performance in marmosets that did not have this initial pretraining and instead were trained directly with the two-position choice phase. Based on data from the present studies, our future training paradigm in behaviorally naïve subjects will include the pretraining component.

For the purpose of these studies, there were no planned statistical analyses (e.g., male vs. female; young vs. aged). This was intentional as an important goal for this set of experiments was to understand the natural variation in the outcome measures, which is essential for identifying appropriate translational model systems and analogous clinical assays before developing hypotheses of how they can be altered: vis a vis in the context of disease. Importantly, these studies provide essential insight and knowledge of natural variation in performance and task acquisition across a population of laboratory marmosets that are not preselected for only high performers. This is especially important for our research program and the ongoing studies in our colony of marmosets genetically engineered with risk variants for AD; every subject in the population is comprehensively characterized irrespective of their performance levels on specific tasks. Relatedly, we are aware that not all subjects will complete all tasks within the battery, and there are many uncontrollable factors across the lifespan of a marmoset (e.g., health issues, breeding, planned veterinary procedures) that may preclude the ability to collect every data point. Therefore, an important component of our research program is not to exclude any subject but rather utilize within-subject analyses longitudinally to track peak performance and subsequent decline and in association with changes in disease biomarkers (e.g., for AD). This approach is much more in line with how human patients are studied. All of these factors (e.g., anxiety, social behaviors, biomarkers, genomics) are being incorporated into our MARMO-AD consortium to understand the divergence of normal aging from disease to improve translational studies from animal models to humans (Sukoff Rizzo et al., 2023).

MARMO-AD was established with funding from the National Institute on Aging (NIA) in line with open science initiatives established in support of the National Plan to Address Alzheimer’s Disease (Khachaturian, Khachaturian & Thies, 2012). The overarching goals of MARMO-AD are to bridge the rodent to human translational gap by conducting comprehensive characterization of both outbred marmosets and marmosets carrying genetic risk factors for AD throughout their lifespan. This will facilitate the study of those mechanisms that drive divergence from nonpathological aging toward inception and pathogenesis of AD. MARMO-AD will integrate multimodal characterization data of the marmosets including assessments of neuropathology (biomarkers), genetics (sequencing), molecular (multi-omics analyses), functional (neuroimaging), behavioral (affective, social), and cognitive assessments as parallels to clinical disease staging and symptoms of AD (Sukoff Rizzo et al., 2023). The consortium aims to take full advantage of the natural genetic and phenotypic diversity in the marmosets, which mirrors that of humans, including individual variation in behavioral responses to enable a better understanding of clinical and biological significance, which is a departure from traditional preclinical approaches that focus on statistical significance (Branch, 1999; Szucs, 2016). As part of this Open Science initiative, all data and protocols will be made available through the adknowledgeportal.synapse.org. The marmoset was specifically selected for this consortium project as it has several advantages over other laboratory animal species as well as other NHPs for the study of aging and aging-related disorders (Perez-Cruz & de Dios Rodriguez-Callejas, 2023; Ross & Salmon, 2019; Sukoff Rizzo et al., 2023). While the development of cognitive touchscreen batteries has been successfully accomplished in macaques (Loyant et al., 2022, 2023; Sorwell et al., 2017; Weed et al., 1999; Wither et al., 2020), only a few labs have reported the successful integration of multiple touchscreen tasks in marmosets within a battery but with limited studies in aging marmosets (Kangas et al., 2016; Sadoun et al., 2019; Spinelli et al., 2004). This development of a comprehensive testing battery addresses several missed opportunities within the aging research field that may contribute to the lack of translational success from animal models to clinical studies. First, the ability to study cognitive development and decline across the lifespan within the same individual; second, the integration of multiple cognitive domains to facilitate a more refined understanding of how pathological aging perturbs the trajectory of typical cognitive aging; and third, data from the battery of cognitive assessments can be integrated with a broad range of tests already established in marmosets that capture negative and positive valence, which are relevant to the spectrum of neuropsychiatric features of aging and AD that are diagnostic for dementia (Granholm et al., 2008; Lyketsos et al., 2011; Oikonomidis et al., 2017). Some of the earliest cognitive changes reported with aging in humans is age-related decline in processing speeds, which is a relative measure from an individual’s peak performance and includes both speed of motor responses and speed at which cognitive activities are performed (reviewed in Salthouse, 2010;Harada et al., 2013 ; Murman, 2015). Age-related memory changes may not only be related to slower speed of processing with respect to recall of information but also rely on domains of attention, including the ability to filter out extraneous stimuli (selective attention) or divided attention, which is the ability to focus on multiple tasks at once (Harada et al., 2013; Murman, 2015). Similarly, our cognitive battery addresses behavioral impulsivity through the 4-CSRTT and cognitive flexibility through evaluation of the ability to switch rules across tasks, which is most robustly emphasized via the switch from DMTP to DNMTP. Changes in processing speed and impulsive behavior in humans with AD are evident during the long prodromal phase that precedes diagnostic mild cognitive impairment, which equates to years of time—relative to weeks within the confines of a short rodent lifespan where only two or perhaps three longitudinal testing batteries may be possible. Importantly, many AD rodent models have demonstrated hyperactivity with aging, thus limiting their translational ability in this respect (Rodgers et al., 2012; Onos et al., 2016). Furthermore, multiple within-subject touchscreen-based tasks conducted longitudinally with aging also is challenging in rodents not only given the food restriction requirement but also the lengthy time periods required to train and assess rodents across each task. In the present studies, while we were limited in evaluating longitudinal changes in our colony across all tasks, we were able to decipher changes in cognitive domains of specific subjects that may influence another. Specifically, subject #21 acquired DMTS training to criteria but was not able to overcome chance levels during the retention memory component of the TU-DMTS test, which was not an issue for subject #2. To understand whether impairments in attention influenced performance on this task, both subjects were evaluated in the 4C-SRTT. Interestingly, subject #21 demonstrated vigilance in attention even at reduced stimulus durations and with speed of processing comparable to subject #2, despite differences in age. Furthermore, subject #21 demonstrated limited spatial working memory capacity as measured by a relatively short delay threshold of 3 s. These data may be indicative of selective impairments in memory for subject #21 independent of impairments in attention, although additional data will be required, including ongoing longitudinal assessments, which are currently in progress for this subject as well as all others in our colony. Moreover, as part of the testing battery, the progressive ratio test assesses motivation; reduced motivation as measured by decreases in breakpoint, may be indicative of anhedonia, an important neuropsychiatric symptom of aging and AD, and which also is assessed for each subject (Granholm et al., 2008; Lyketsos et al., 2011; Oikonomidis et al., 2017). While indeed the primary symptom of AD is traditionally cognitive decline and memory loss, there are also other non-cognitive aging-related changes in mood as well as alterations in sleep, anxiety, depression, apathy, and social withdrawal which may also be exacerbated or have an accelerated progression of change as a consequence of disease (Granholm et al., 2008; Masters et al., 2015). The sophisticated social and emotional behavioral repertoire of the marmoset and the planned longitudinal lifespan studies described herein will provide the opportunity to study progression of such changes with aging, which is an important component of the translational studies planned as part of MARMO-AD (Sukoff Rizzo et al., 2023).

On balance, the extensive time and resource investment to train marmosets across a battery of tasks is significant. As described above, and can be inferred from the detailed results indicating days to criteria for each task, marmosets show substantial individual variability during initial training to meet task criterion for each test. We would argue that the initial training time invested in a battery, as opposed to a single test per subject is warranted, especially as described above in the context of deciphering how performance in one cognitive domain on a specific task may be influenced by that of another (e.g., attention vs. working memory or motivation). Importantly, although the initial training time for the battery may exceed 6 months, many of the initial training steps are not required for longitudinal testing such that retesting can be reduced to just weeks. More specifically, longitudinal retesting of the tasks described herein is estimated as approximately 8–12 weeks annually. That being said, because the present studies are planned as lifespan studies, habituation to new training staff members throughout the lifespan of the marmoset may extend training and testing times, which are marmoset-dependent. Recently, new automated, unsupervised technology has been used for cognitive training in marmosets, which may aid in facilitating training and potentially reduce subject habituation to new staff members (Calapai et al., 2022). We caution that automated systems still require step-wise training to a priori criterion by trained staff with knowledge and expertise in behavioral and cognitive testing and ideally aided by cameras to ensure that responses are targeted and paired with rewards. This initial training was appreciated by Calapai and colleagues before deploying the unsupervised system (Calapai et al., 2022). Importantly, as with any automated system, should the unsupervised instrument fail during a critical training step or a stressful event occurs while the marmoset is engaged with the system, this could result in behavioral extinction or a negative association resulting in poor performance which could potentially be misinterpreted as cognitive impairment. Relatedly, it cannot be ruled out that decreased performance in unsupervised systems in the absence of discreet, timed sessions could be potentially influenced by satiety. Thus, while there may be benefits of using automated training systems, the potential challenges and limitations in addition to costs and limited distribution of these systems for testing to date have minimized our enthusiasm at this time. Furthermore, while food and/or water deprivation before testing also has been suggested to facilitate task acquisition in marmosets (Spinelli et al., 2004) within the present studies, the intent was to design testing in such a manner that food deprivation would not be required by instead identifying salient rewards. Given that the nature of our studies as longitudinal throughout lifespan, including during periods of advanced aging, disease (e.g., Alzheimer’s-related pathologies), and potentially during pregnancy, we did not investigate the potential benefits of food or water restriction to accelerating task acquisition. Given that all but one marmoset of the 34 marmosets successfully acquired the touch-reward association and completed the initial touch training in the absence of requiring any food restriction with as few as eight training sessions, we believe that preferred rewards may have similar salience to that of restriction without the additional potential stressor.

While as pointed out, the marmoset as a model system for the study of aging and aging-related disorders, including cognitive trajectories has many benefits, it is important to emphasize limitations. For example, key brain pathologies related to aging and aging disorders, which have been studied in relationship to cognitive decline across NHP species, have been much better characterized in macaques. More specifically, while multiple labs have reported aging-related amyloid plaque deposition in both marmosets and macaques, the presentation of neurofibrillary tau tangles (NFT), which have been proposed to correlate with cognitive decline in humans, is rare and sporadic in aged marmosets (Rodriguez-Callejas, Fuchs, & Perez-Cruz, 2016; Arnsten et al., 2021). In contrast, macaques present with highly conserved tau pathology with aging, consistent with Braak staging in humans (Arnsten et al., 2021; Papalas et al., 2018). The limited studies of aging-related presentations of NFTs in marmosets may be confounded by their shorter lifespan in captivity, although experimental approaches, including engineering of genetic risk factors into the marmoset as well as exogenous tau seeding studies, are in progress to better understand tau progression in marmosets (Sukoff Rizzo et al., 2023). Another limitation of marmosets is their lack of reproductive senescence with aging, which is an important area of study related to dementia and cognitive decline. The emergence of dementia and Alzheimer’s disease after menopause has fostered hypotheses that estrogen may serve as a neuroprotective factor potentially through epigenetic mechanisms (Conde et al., 2021; Pertesi et al., 2019; Ratnakumar et al., 2019). As marmosets only exhibit ovarian aging through anovulation associated with follicular depletion very late in their life, studies of estrogen depletion and cognitive decline may be better suited for macaques or other NHP species (Tardif et al., 2011; Walker & Herndon, 2008).

## Conclusions

The present studies provide insight toward a paradigm-shifting, “*marmoset as the patient*” approach, which integrates longitudinal cognitive testing throughout the lifespan with other behavioral, pathophysiological, and genomic data. Together this approach will provide robust datasets similar to those being collected in patients that can be used to identify mechanisms that precede cognitive decline and can be identified as targets for therapeutic interventions. While these are the initial data that have been generated to inform larger-scale projects, these studies were critical in providing insight and foundational knowledge to allow for iterations in the testing battery before enrolling new cohorts that will be tested from adolescence throughout their lifespan. We believe the detailed methods and approaches shared here, including emphasizing and incorporating the inherent individual variability of genetically heterogenous marmoset populations, are likely to be broadly generalizable for other studies evaluating cognitive function in marmosets beyond aging and AD.

## Data Availability

Data and methods are available through the AD Knowledge Portal: https://adknowledgeportal.synapse.org/Explore/Programs/DetailsPage?Program=MARMO-AD.

## Code Availability

(software application or custom code): The Touchscreen hardware and software (ABET) used for these studies are commercially available products purchased through Lafayette. The custom fabrication of the touchscreen training box design details and specific code written for each task are available by request.
